# Primary intraosseous carcinoma arising from an odontogenic cyst: A case report

**DOI:** 10.3892/ol.2014.2248

**Published:** 2014-06-12

**Authors:** MAKOTO ADACHI, TOSHIHIRO INAGAKI, YUICHI EHARA, MUNEHIRO AZUMA, AYUMI KURENUMA, MASAYUKI MOTOHASHI, YASUNORI MURAMATSU

**Affiliations:** Department of Oral and Maxillofacial Surgery, Asahi University Murakami Memorial Hospital, Gifu 500-8523, Japan

**Keywords:** intraosseous carcinoma, odontogenic cyst, mandible, jaw tumor

## Abstract

Cyst-like lesions in the mandible rarely develop into malignancies, and the reported incidence is between 0.3 and 2%. The present study describes a rare case of primary intraosseous squamous cell carcinoma of the mandible arising from an odontogenic cyst. A 59-year-old female was referred to Asahi University Murakami Memorial Hospital (Gifu, Japan), with acute pain in the right molars. An initial examination revealed buccal swelling and paresthesia of the mental nerve. Following an intraoral examination, the oral mucosa was confirmed to be normal, however, percussion pain was experienced between the lower right first premolar and second molar. Panoramic radiography revealed a retained lower right wisdom tooth and an irregular radiolucent area between the lower right molar and a mandibular angle with unclear margins. Computed tomography revealed diffuse bone resorption and an extensive loss of cortical bone on the buccal and lingual sides. A biopsy was performed and the pathological diagnosis was of a squamous cell carcinoma arising from the epithelial lining of the odontogenic cyst. Radical dissection was subsequently performed, however, histopathological examination of the resected specimen revealed neither invasion into the surrounding tissues penetrating the periosteum nor lymph node metastasis at the right submandibular lesion. Following the pathological diagnosis of primary intraosseous carcinoma (PIOC), the patient received 6,000 Gy radiation as post-operative radiotherapy and chemotherapy with oral administration of tegafur, gimeracil and oteracil potassium. The patient is currently undergoing follow-up examinations. Although PIOC arising from an odontogenic cyst is rare, it should be considered as a differential diagnosis for radiolucency of the jaw bone, particularly in older patients exhibiting a history of cystic lesions.

## Introduction

Primary intraosseous carcinoma (PIOC) of the jaw is a rare carcinoma arising from the odontogenic epithelium without connection to the oral mucosa. The tumor is considered to develop from a remnant of the odontogenic epithelium.

PIOC was first described as a central epidermoid carcinoma of the jaw by Loos (1) in 1913. The classification of PIOC has since undergone numerous revisions and the precise diagnostic criteria of PIOC has not yet been established.

The term PIOC was established by the World Health Organization (WHO) as part of the classification for the histological typing of odontogenic tumors (2). Elzay (3) reviewed the literature associated with PIOC of the jaw and subsequently insisted that a modification of the WHO classification was required. Slootweg and Müller (4) also recommended modifications, while a study by Waldron and Mustoe (5) and Müller and Waldron (6) added intraosseous mucoepidermoid carcinoma to the classification.

According to the WHO (7), PIOC may be categorized into three types: i) A solid tumor invading the bone marrow spaces and inducing osseous resorption; ii) a squamous cell carcinoma arising from the epithelial lining of an odontogenic cyst; and iii) a squamous cell carcinoma that is associated with other benign epithelial odontogenic tumors.

Although the classification has improved, the etiology of PIOC remains unclear. PIOC may be derived from the direct transformation of the odontogenic epithelium, particularly the odontogenic epithelial rests, such as Malassez’s epithelial rest, from within the alveolar bone following tooth loss or from the remnants of the dental lamina and the reduced enamel epithelium surrounding an unerupted or impacted tooth (8).

With regard to the rare cases of PIOC derived from odontogenic cysts, a common criteria for PIOC has been established based on the published case reports and is as follows: i) The absence of another primary tumor on chest radiographs, as metastatic carcinoma is the most common malignancy of the jaw and thus, the diagnosis of PIOC must always be confirmed by the exclusion of a metastasis; ii) the absence of an ulcer in the oral mucosa overlaying the tumor; and iii) histopathological evidence of transition of the epithelial lining into squamous cell carcinoma (9,10).

In the current study, a case of PIOC arising from an odontogenic cyst is presented and the issues concerning the differential diagnosis and management are discussed. The patient provided written informed consent.

## Case report

A 59-year-old female was referred to Asahi University Murakami Memorial Hospital (Gifu, Japan), with acute pain in the right molars. There was no history of tobacco or alcohol use, however, the patient had suffered from hyperlipidemia several years previously.

**Figure 1:**
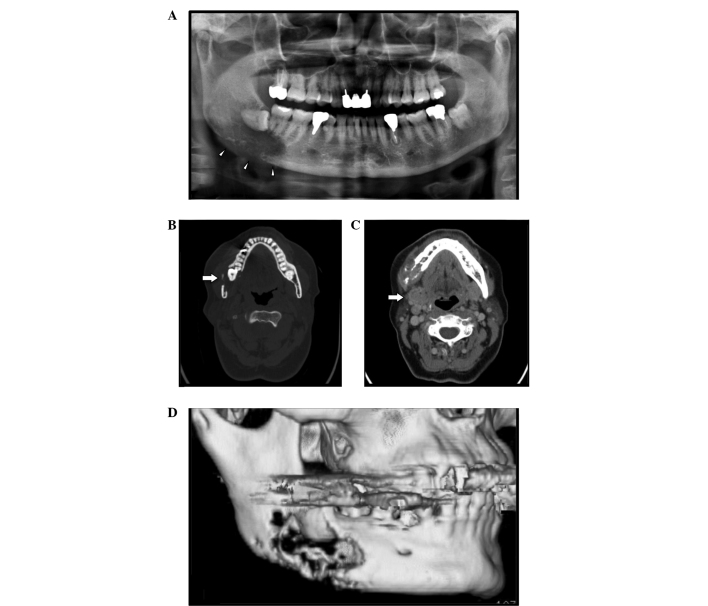
(A) Panoramic radiography of the patient showing a retained lower right wisdom tooth and an irregular radiolucent area between the lower right molar and mandibular angle with unclear margins (arrowheads). (B) Axial computed tomography (CT) revealing a large oval mass of 44×31×35 mm at the right angle of the mandible between the second premolar and ramus (arrow), with extensive bony destruction of the lingual and buccal cortex. (C) Enhanced axial CT revealing pathological lymph node enlargement at the right submandibular lesion (arrow). (D) Three dimensional CT scan revealing an osteolytic lesion, which perforated the lingual and buccal cortex with mandibular canal involvement.

Initial observations revealed right buccal swelling and paresthesia of the mental nerve. An intraoral examination revealed a normal oral mucosa, however, percussion pain was experienced between the lower right first premolar and second molar. The routine panoramic radiograph showed a retained lower right wisdom tooth and an irregular radiolucent area between the lower right molar and mandibular angle, with unclear margins (Fig. 1A). In addition, computed tomography (CT) revealed a large oval mass, 44×31×35 mm in size, at the right angle of the mandible between the second premolar and ramus, with extensive bony destruction of the lingual and buccal cortex and pathological lymph node enlargement at the right submandibular lesion (Fig. 1B–D).

Laboratory results revealed that the white blood cell count was 5,400 cells/μl and the C reactive protein level was 0.3 mg/dl.

Following a biopsy of the lesion, squamous cell carcinoma arising from an epithelial lining of an odontogenic cyst was diagnosed. Two weeks after diagnosis, radical surgery (a hemi-mandibulectomy with primary suture and reconstruction using a titan reconstruction plate and modified radical neck dissection) was performed under general anesthesia.

**Figure 2:**
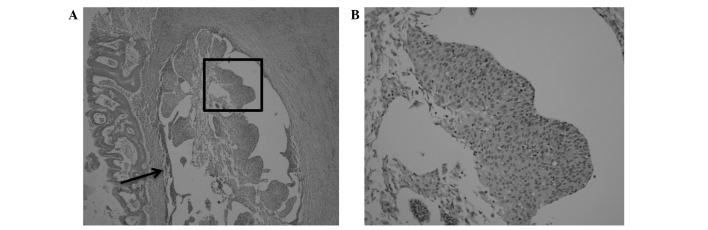
Photomicrographs of the lesion obtained from the surgical specimen revealing moderately-differentiated squamous cell carcinoma (framed area), with intact squamous epithelium presented by the arrow, which was observed to be overlying the tumor. Hematoxylin and eosin stain; magnification, (A) ×40 and (B) ×200 (framed area).

Intraoperative observations revealed that the tumor had extended through the buccal and lingual cortex and invaded the masseter and internal pterygoid muscles. In addition, pathological examination of the surgical specimen revealed squamous cell carcinoma with an intact squamous epithelium, which was observed to be overlying the tumor (Fig. 2). Lymph node metastasis was not observed in the neck lymph nodes. A positive margin was present in the specimen at the end of the inferior alveolar nerve, therefore the patient received post-operative radiotherapy, and chemotherapy. External beam irradiation was performed five times per week at 2 Gy per fraction to a total of 60 Gy, while the doses of the oral administration of tegafur, gimeracil and oteracil potassium were 60 mg/m^2^/day for two weeks followed by a two week rest for a total of six months. The one-year post-operative follow-up revealed no local recurrence or distant metastasis.

## Discussion

PIOC is a rare carcinoma that arises from the direct transformation of odontogenic epithelial rests in the jaw, including the epithelial rests found within the alveolar bone and periodontal ligament, a persistent dental lamina and the enamel epithelium surrounding an impacted tooth (7,8).

The factors responsible for the malignant transformation of the cystic lining of odontogenic cysts remains unclear. The most common factor may be a chronic inflammatory stimulus with or without a predisposing genetic cofactor, which induces neoplastic transformation (11,12).

Coussens and Werb proposed a potential association between sites of chronic inflammation and the development of cancer as early as 1863, and this has proven to be fairly accurate (13). A number of cancers have been presumed to originate in tissues that are chronically inflamed, and the inflammatory microenvironment is considered to promote the progression of malignancy, including initiation, growth, angiogenesis, invasion and metastasis, however, the precise mechanisms have not yet been established (5).

PIOC occurs in a wide age range of individuals, having been identified in patients between 1.3 and 90 years, with an estimated mean age of 60.2 years. Furthermore, the incidence of PIOC is much higher in males than females (14).

PIOC has most frequently been found in the molar-ramus region of the mandible (15,16). The recurrent clinical symptoms are swelling, pain/toothache and lesion growth. These early symptoms are commonly followed by trimus and numbness of the mandibular nerve and muscle invasion. In the present case, the patient exhibited right buccal swelling and paresthesia of the mental nerve.

Although the diagnostic criteria of PIOC remains unclear, the following criteria have been suggested: i) The tumor must be a histopathologically-based squamous cell carcinoma without the involvement of any other odontogenic cysts or metastatic tumor cells; ii) it must exhibit intact mucosa; and iii) no other distant primary tumor must be present at the time of diagnosis, with at least a six-month absence of malignancy during the follow-up period (9). The patient in the present case met all of these criteria.

Radiographical examination is an effective method for the diagnosis of PIOC. PIOC usually exhibits marked variation in the appearance of its border (17), and thus, it is worth considering as a differential diagnosis of jaw radiolucency. While panoramic radiography is useful for obtaining an overall view of the disease, it may be limited by not providing an evaluation of bone destructive lesions with a ragged border, the margin, or the degree of extension and invasion of the surrounding tissue of the tumor mass. Similarly, panoramic radiography does not sufficiently show soft masses. In certain cases, PIOC mimicks periapical and periodontal lesions, which leads to misdiagnosis (9). CT provides detailed information regarding the location, size and shape of the lesion, and allows for the visualization of the cited feature as an indicator of the aggressiveness that is common in cases of potentially malignant maxillofacial tumors (18,19).

Radical surgery with adequate resection appears to be the most significant factor in the successful treatment of PIOC (20). Without an initial biopsy, the radiolucent lesion may simulate an odontogenic cyst, leading to the enucleation of the lesion without adequate free margins. Thus, for the correct treatment, a histopathological diagnosis must be made during the surgical intervention. For metastatic lymph node lesions, a neck dissection may be recommended in cases of PIOC arising from odontogenic cysts. Suspected lymph node metastasis prior to surgery requires a block dissection with the primary lesion (6,16). Radiotherapy and chemotherapy are only used as palliative therapy, or as adjuvant therapy in cases where nerve infiltration is diagnosed (20). In the present case, the tumor was found to extend along the inferior alveolar nerve and thus, the patient received subsequent post-operative radiotherapy.

The present study documents a case of PIOC arising from an odontogenic cyst. Due to its rarity, it should be considered as a differential diagnosis of radiolucency of the jawbone, particularly in older patients with a history of cystic lesions in the jawbone. Not only is a biopsy recommended, but also the removal of the entire cyst wall, since malignant changes in the epithelial lining may not be visible in all sections of the lesion. The validity of this recommendation is supported by the low rate of regional lymph node metastasis, with only six reported cases and the poor survival rate reported for these patients (14). Future studies must strictly adhere to a well-delineated classification that will thus provide further comparative and confirmatory data on PIOC.
